# The Treatment of Infected Wounds

**Published:** 1919

**Authors:** 


**The Treatment of Infected Wounds.** By A. CARREL AND G. DEHELLY. Translation by HERBERT CHILD. Second Edition. Pp. x., 265. London : Baillière, Tindall & Cox. 1918.

Many surgeons have been led by the war to modify their former view of the value of the ordinary antiseptics in infected wounds, and the treatment evolved by M. Carrel in the use of hypochlorite of soda solution is one of the " discoveries " of military surgery.

Whatever view we choose to take of the explanation of the antiseptic efficiency of Carrel's method, the fact remains that he has shown that infected wounds—early or late—can be sterilised by the means he has devised, and no greater tribute can be paid to the successful results so obtained than the words of a printed notice which the writer of this review saw on the walls in M. Tuffier's wards in Paris, where the Carrel treatment was in use : " *Tout blessé qui suppure a le droit d'en demander la raison à son chirurgien.*"

The present volume is a full exposition of the technique described by the originator of this form of treatment, and on comparing the English with the French Edition we can say that the translator has done his work well. The text is elucidated by a considerable number of illustrations.

